# Silencing of DNase Colicin E8 Gene Expression by a Complex Nucleoprotein Assembly Ensures Timely Colicin Induction

**DOI:** 10.1371/journal.pgen.1005354

**Published:** 2015-06-26

**Authors:** Simona Kamenšek, Douglas F. Browning, Zdravko Podlesek, Stephen J. W. Busby, Darja Žgur-Bertok, Matej Butala

**Affiliations:** 1 Department of Biology, Biotechnical Faculty, University of Ljubljana, Ljubljana, Slovenia; 2 Institute of Microbiology and Infection, School of Biosciences, University of Birmingham, Birmingham, United Kingdom; Uppsala University, SWEDEN

## Abstract

Colicins are plasmid-encoded narrow spectrum antibiotics that are synthesized by strains of *Escherichia coli* and govern intraspecies competition. In a previous report, we demonstrated that the global transcriptional factor IscR, co dependently with the master regulator of the DNA damage response, LexA, delays induction of the pore forming colicin genes after SOS induction. Here we show that IscR is not involved in the regulation of nuclease colicins, but that the AsnC protein is. We report that AsnC, in concert with LexA, is the key controller of the temporal induction of the DNA degrading colicin E8 gene (*cea8*), after DNA damage. We demonstrate that a large AsnC nucleosome-like structure, in conjunction with two LexA molecules, prevent *cea8* transcription initiation and that AsnC binding activity is directly modulated by L asparagine. We show that L-asparagine is an environmental factor that has a marked impact on *cea8* promoter regulation. Our results show that AsnC also modulates the expression of several other DNase and RNase colicin genes but does not substantially affect pore-forming colicin K gene expression. We propose that selection pressure has “chosen” highly conserved regulators to control colicin expression in *E*. *coli* strains, enabling similar colicin gene silencing among bacteria upon exchange of colicinogenic plasmids.

## Author Summary

Colicins are considered model proteins for studying bacterial toxins. These narrow spectrum antibiotics can kill by a variety of mechanisms, e.g. by forming pores in the membranes of susceptible cells or by degrading their nucleic acids. Colicin genes are plasmid-encoded and repressed by the master regulator of the DNA damage response, LexA. Induction of several pore-forming colicin genes is also repressed by IscR, which ensures that colicin genes are switched on as a last resort in DNA damaged cells, when nutrients are depleted. Here we show that nuclease colicin genes are not controlled by IscR but that the AsnC protein, in concert with LexA, is directly responsible for uncoupling the immediate expression of the DNase colicin E8 from the main induction of the SOS response. AsnC wraps the DNA of the colicin E8 promoter into a complex nucleoprotein assembly and the architecture of this complex is altered by the presence of the amino acid L-asparagine. Thus, repression by metabolite-responsive and DNA-damage responsive regulators operates at the regulatory regions of different colicins. Hence, the response to several environmental signals have been integrated to ensure that, following DNA damage, colicin synthesis is tightly repressed and induced only in terminally damaged cells.

## Introduction

Colicins are high-molecular-weight toxic proteins that are produced by and specifically target *Escherichia coli* and its close relatives [1]. These narrow-spectrum antibiotics kill by either targeting the DNA, RNA or cell membranes of susceptible cells. Cytoplasmic colicins are released upon the synthesis of a lysis protein, the expression of which is independent of intracellular colicin accumulation [2]. This causes the stochastic lysis of producing cells and is suggested to assist surviving sister cells by killing potential competing sensitive cells [3]. Colicin-mediated competition has been suggested to have functions in modulating population dynamics and maintaining diversity of microbial communities [4–7]. Nutrient limitation and DNA damage seem to be the major signals that control colicin production, enabling interference competition among strains [8].

Colicins are plasmid-encoded and are expressed from strong promoters whose activity is tightly repressed by the LexA transcription factor, the master regulator for the SOS DNA damage repair response in bacteria [1,9]. Most of the SOS genes involved in DNA repair and cell division arrest are expressed immediately after DNA damage, but induction of colicin genes is delayed. This presumably provides cells time to repair DNA in order to preserve the integrity of their genome, before the induction of colicin production [10]. In previous work, we established that the global transcriptional repressor IscR delays the induction of the pore-forming colicin K gene (*cka*) [11]. We showed that IscR participates in a double-locking mechanism, in concert with LexA, by stabilizing the LexA SOS repressor at the promoter and this links colicin expression to the nutritional status of the cell. Thus, the IscR protein uncouples the induction of colicin expression from the temporal induction of the SOS response that deals with repairable DNA damage. This mechanism also operates at other promoters, which control the expression of bactericidal pore-forming colicins [11], however, it is not known if a similar fail-safe double-lock system has also evolved for the nuclease colicin genes.

Here we report that IscR does not modulate the expression of nuclease colicin genes. Hence, we studied the regulation of the DNA degrading colicin E8 gene (*cea8*) in more depth and identified the AsnC transcription factor as directly responsible for the delay in *cea8* expression. AsnC is a member of Lrp/AsnC family of transcriptional regulators that modulate cellular metabolism in both archaea and bacteria [12,13]. In *E*. *coli*, AsnC is required to activate the expression of the L-asparagine synthetase A gene (*asnA*) and this stimulation is abolished in the presence of the amino acid L-asparagine [14]. In addition to this, expression of *asnC* is negatively autoregulated by AsnC and also repressed by the nitrogen assimilation control (Nac) protein, under nitrogen-limiting conditions [15], however, this regulation is not modulated by the presence of L-asparagine [14]. Functional *E*. *coli* AsnC is an octamer, whose structure was resolved by X-ray crystallography [13]. We show that AsnC binds to the *cea8* regulatory region at multiple sites, likely wrapping the DNA into a nucleoprotein assembly and that its binding is affected by L-asparagine. In the LexA-AsnC-*cea8* complex, two LexA dimers are flanked by multiple AsnC octamers, and the presence of L-asparagine influences AsnC modulated promoter region geometry. Data presented here shows that double locking by LexA and AsnC operates at the *cea8* promoter region to delay induction of the colicin E8 gene, thereby linking *cea8* expression to DNA damage and L-asparagine availability. Thus, AsnC provides colicinogenic cells with time for DNA damage repair and limits colicin E8 induction to terminally damaged cells.

## Results and Discussion

### IscR does not regulate the induction of the DNA or RNA degrading colicins

**Fig 1:**
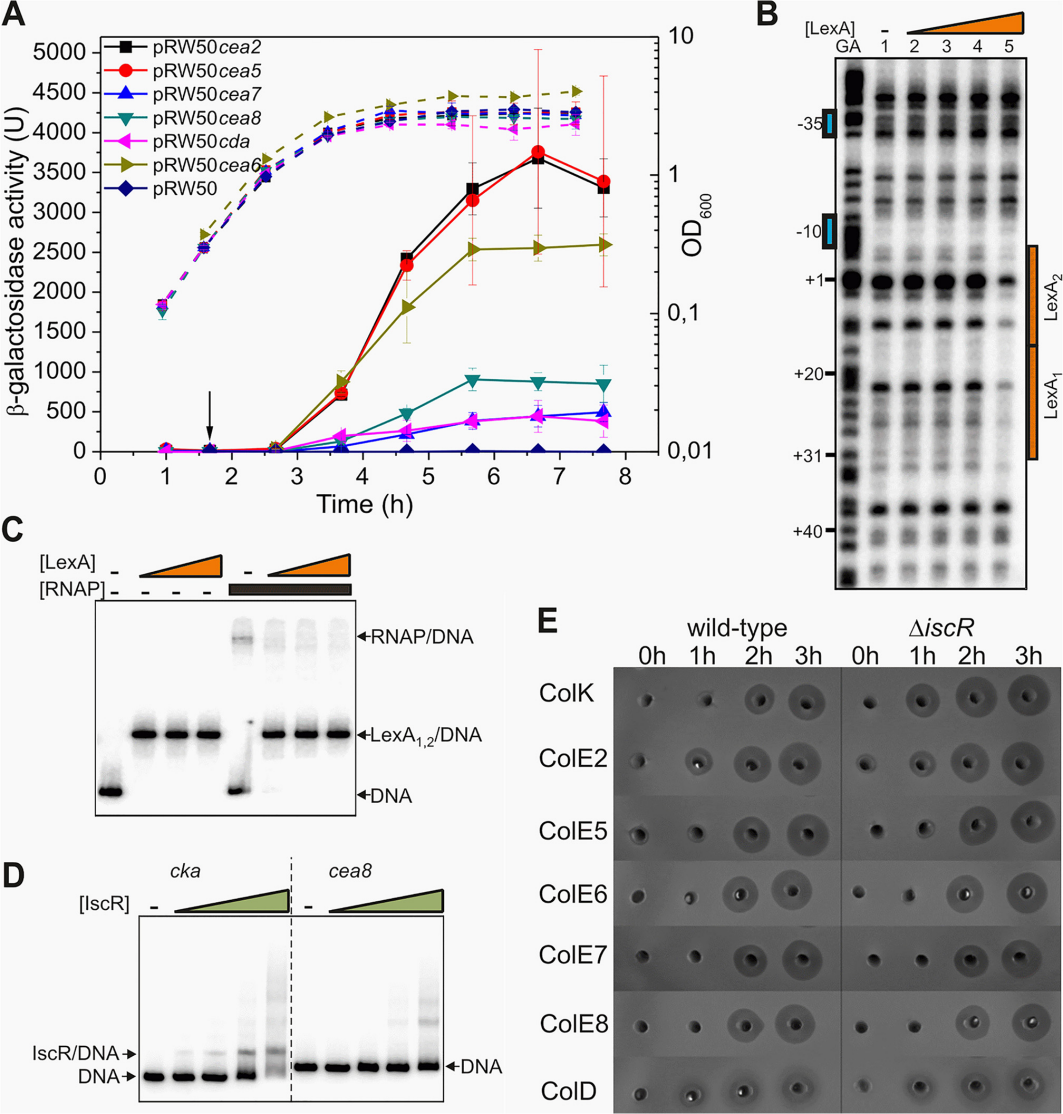
IscR does not modulate the temporal induction of nuclease colicins. A) Measured β-galactosidase activities of strain BW25113, carrying the *lac* expression vector pRW50 containing various nuclease colicin promoter fragments. Each value represents the mean ± SD of at least three independent measurements, the arrow indicates the time of addition of a sub-lethal concentration of nalidixic acid (37 μM) and the dashed lines represent optical density (OD_600_). B) The binding of purified LexA protein to a P^32^ end-labelled *cea8* fragment was investigated using DNase I footprint analysis. The location of the two LexA binding sites, LexA_1_ and LexA_2_ is indicated by orange boxes and the -10 and -35 promoter elements by blue boxes. The final concentration of LexA protein used in reactions was 12.5, 25, 50 and 100 nM (lanes 2–5). C) The binding of purified LexA and RNA polymerase (RNAP) to a P^32^ end-labelled *cea8* fragment was investigated using EMSA analysis. DNA fragments were first incubated for 15 min with various concentrations of LexA (100, 200 or 400 nM) followed by the addition of 100 nM RNAP with further incubation for 15 min at 37°C. The location of free DNA, the LexA/DNA and RNAP/DNA complexes is indicated. D) The binding of purified IscR protein to P^32^ end-labelled colicin K (*cka*) or *cea8* promoter fragments was assayed using EMSA analysis. The concentration of IscR used was 0.4, 0.8, 1.6 and 3.2 μM. The location of free DNA and the IscR/DNA complex is marked. E) Assays of colicin production in BW25113 and its Δ*iscR* derivative cells, carrying various colicin-encoding plasmids. Equal amounts of cells were collected at hourly time points after the addition of nalidixic acid (0 h) and crude cell extracts were placed into wells in agar plates overlaid with soft agar, harbouring the *E*. *coli* K-12 indicator strain DH5α pBR322. Experiments were performed in duplicate and representative growth curves are shown in S2 Fig.

To study the induction of various nuclease colicins after DNA damage, we assayed the activity of colicin promoters in *E*. *coli* K-12 strain BW25113. In this experiment, different DNA fragments, carrying colicin promoters, were cloned into the *lac* expression vector, pRW50, to give colicin promoter::*lac* fusions. After inducing the DNA damage response with a sub-inhibitory concentration of nalidixic acid, we observed that the promoters of the DNA degrading colicins E2, E7 and E8, of colicins E5 and D, targeting tRNA, and of the rRNA cleaving colicin E6, are only induced after a prolonged delay (Fig 1A). Note that there is little expression from nuclease colicin promoters in the absence of DNA damage (S1 Fig).

We previously established that the delayed expression of several pore-forming colicins, is due to co-repression by the global transcriptional repressors LexA and IscR [11]. At the colicin K promoter, the LexA repressor was shown to bind to the tandem operators just downstream of the -10 promoter element and prevented RNA polymerase binding. The IscR protein was suggested to increase stability of LexA at these targets. To determine if a similar mechanism controls the expression of the nuclease colicins, we investigated the regulation of the DNA degrading colicin E8 by electrophoretic mobility-shift assays (EMSA) and DNase I footprinting. Our results reveal that at the promoter region of *cea8*, the LexA repressor binds to two overlapping targets and blocks the access of RNA polymerase to the promoter (Fig 1B and 1C). To investigate the possible binding of IscR to the *cea8* promoter, an EMSA assay was again used. Results in Fig 1D show that IscR binds specifically to the *cka* promoter region but not to that of *cea8*. In addition, we tested whether the IscR protein is directly responsible for the delayed production of colicin E8 and other nuclease colicins, by comparing the colicin production in our wild-type and Δ*iscR* strains. Results, illustrated in Fig 1E, show that IscR has a negligible effect on the synthesis of many nuclease colicins. This contrasts with the pore-forming colicin K, where the Δ*iscR* allele causes a 100 increase after the first hour of induction (S3 Fig) [11]. This indicates that IscR does not regulate any of the nuclease colicin genes and prompted us to search for other transcription factors, involved in controlling the timing of their expression.

### AsnC delays *cea8* expression

**Fig 2:**
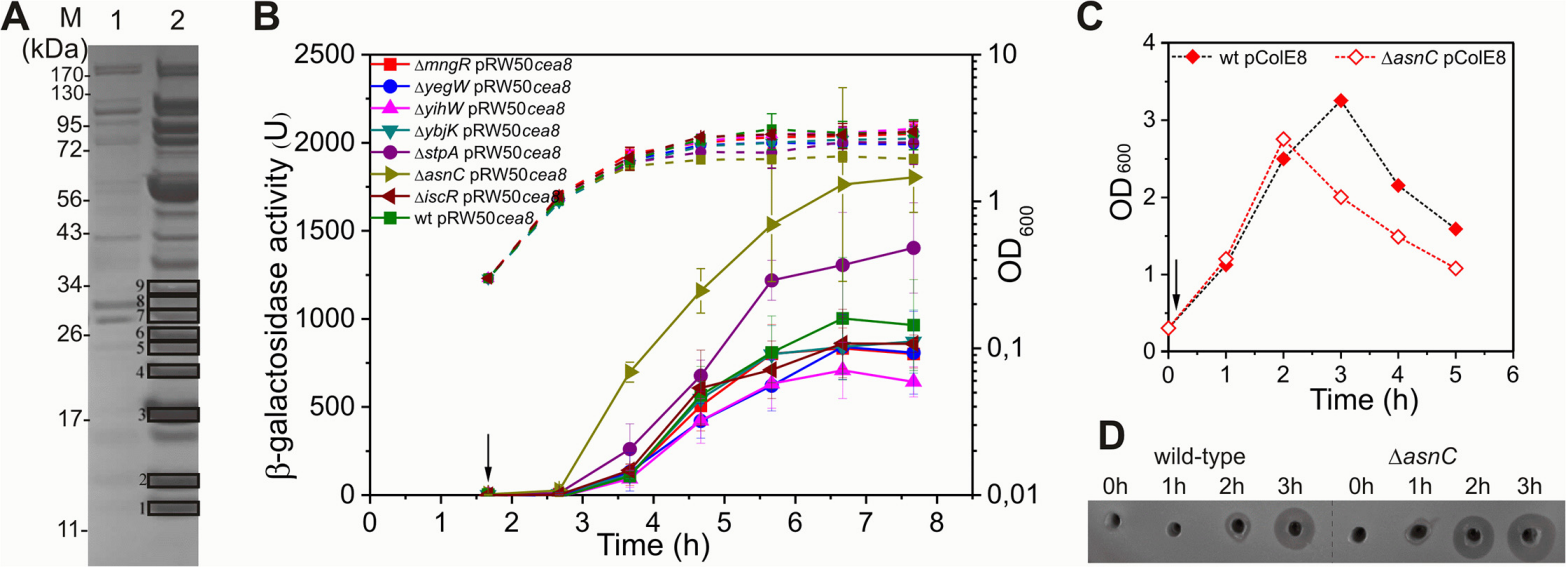
AsnC delays *cea8* expression. A) A Coomassie blue stained SDS-PAGE protein gel, which shows the protein profile of the eluates obtained from a control (lane 1) and *cea8* affinity chromatography (lane 2) experiment. Proteins 1–9, which are denoted by boxes, were trypsin digested and analysed by mass spectrometry. Candidate proteins identified by affinity chromatography are listed in S2 Table. B) The *cea8* promoter region was cloned into the *lac* expression vector pRW50 and transformed into *E*. *coli* strain BW25113 (wt) and various mutant derivatives. The arrow indicates the time of addition of nalidixic acid (37 μM) and the dashed line represents optical density (OD_600_). β-galactosidase activity was determined at hourly intervals. Each value represents the mean ± SD of at least three independent measurements. C) Representative growth curves of BW25113 (wt) and Δ*asnC* cells, carrying plasmid pColE8, which naturally expresses the DNA degrading E8 colicin. The arrow indicates the time of addition of nalidixic acid. D) Assays of colicin production in BW25113 and Δ*asnC* cells, carrying the pColE8. Equal amounts of cells were collected at hourly time points after the time of addition of nalidixic acid (0 h), indicated by an arrow in panel C, and crude cell extracts were applied into wells in agar plates overlaid with soft agar, harbouring the colicin sensitive strain DH5α pBR322. All experiments were performed in duplicate.

To investigate the delay in *cea8* induction in SOS-induced cells we used a pull-down assay [11], using a cleared cell extract from mid-logarithmic grown, SOS induced, *E*. *coli* cells, and a biotinylated 179 bp *cea8* promoter fragment as a bait. Eluted proteins were separated by SDS-polyacrylamide gel electrophoresis and nine bands were analysed by mass spectroscopy (Fig 2A). We identified 30 transcription regulators and nucleoid associated factors that had associated with the bait (S1 Table). To screen for their ability to regulate *cea8* expression after DNA damage induction with nalidixic acid, we measured *cea8*::*lac* activity in deletion mutants from the Keio collection [16] and we selected strains in which a 3-fold increase in *cea8* promoter activity, in comparison to the wild-type strain, was observed (S2 Table). Thus, we focused on the AsnC, StpA, OmpR, YbjK, YihW, YegW and MngR proteins and measured *cea8* promoter activity following SOS induction using pRW50 *cea8*::*lac* fusion in the corresponding deletion mutant strains throughout the bacterial growth curve. Results presented in Fig 2B show that disruption of *asnC* resulted in the biggest effects on *cea8* promoter induction after DNA damage. An intermediate increase in promoter activity was observed in the strain deficient for *stpA*, whilst the other deletions had a minimal effect, with our data confirming that IscR does not regulate colicin E8 expression. The StpA protein, a paralogue of the nucleoid-associated protein H-NS, forms a rigid filament along DNA, and can cause DNA bridging [17]. Furthermore, StpA can act as an RNA chaperone [18] and a transcriptional repressor [19,20], thus, it may be involved in colicin gene expression. However, here we focused on AsnC, and assayed its binding to *cea8* promoter region and its effect on colicin E8 synthesis. To do this, we introduced the Δ*asnC* allele into a strain that harbours a *cea8*-encoding plasmid. After treatment of cells with a subinhibitory concentration of nalidixic acid that induced DNA damage, cell growth and colicin production was compared in the wild-type and the Δ*asnC* mutant. Our results show that AsnC enhances viability of the strain harbouring the colicin E8-encoding plasmid (Fig 2C). Bioassays were also used to follow colicin levels in crude cell extracts prepared from cells before and after SOS induction. The results show that, in the Δ*asnC* strain, colicin E8 was produced an hour earlier in comparison to the delayed synthesis in the wild-type strain (Fig 2D). This suggests that AsnC directly represses *cea8* promoter activity and, in concert with LexA, ensures regulated and delayed expression of the *cea8* gene.

### L-asparagine affects interaction of AsnC with *cea8* promoter region

**Fig 3:**
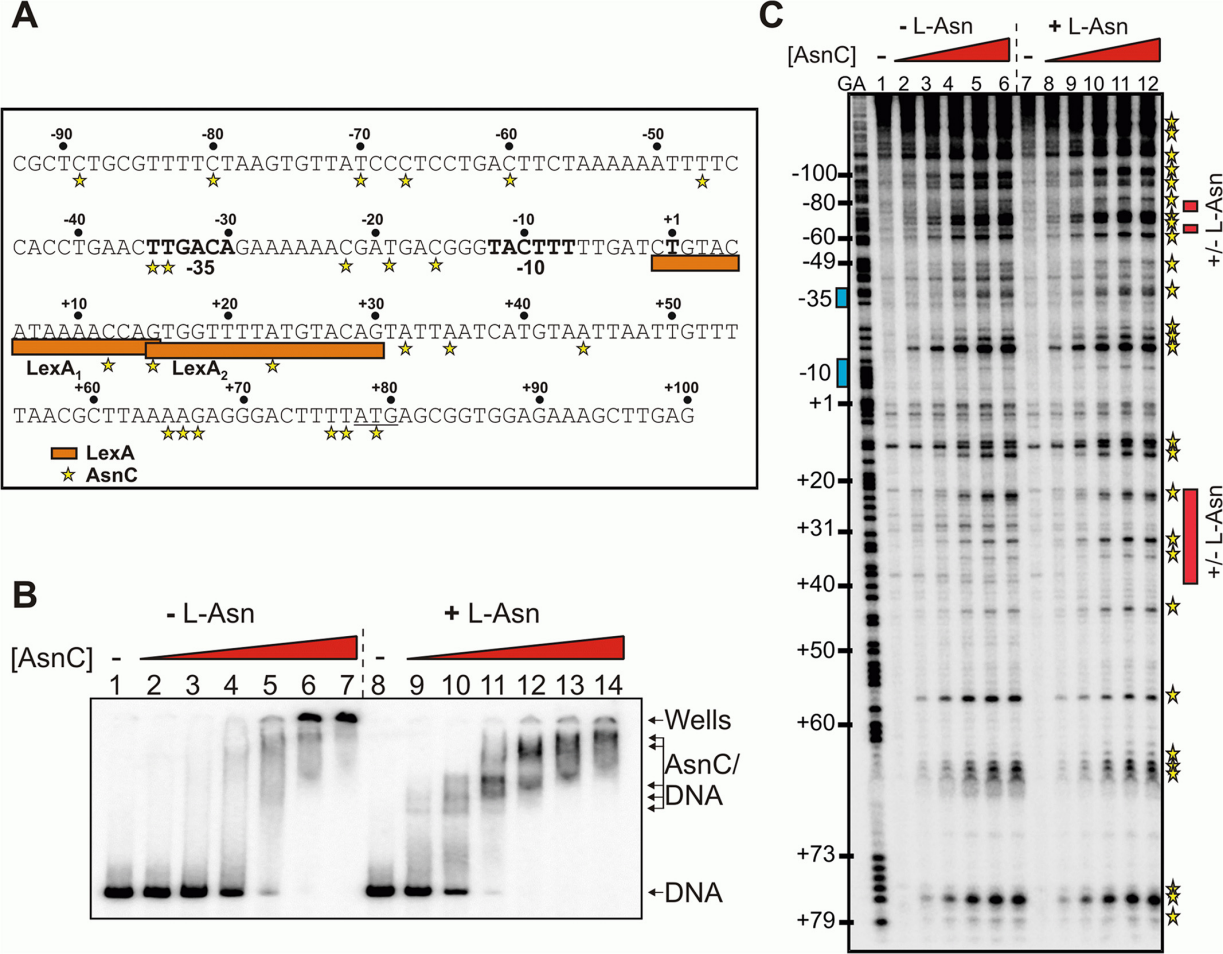
Binding of AsnC protein to *cea8* is altered by L-asparagine. A) Organisation of the colicin E8 promoter region. The figure shows the DNA sequence of the *cea8* promoter from position -93 to +100. The -35 and -10 core promoter elements and the predicted start of transcription (+1) are shown in bold type and the start of translation (ATG) is underlined. The location of the two LexA binding sites (LexA_1_ and LexA_2_) is shown by orange boxes. The AsnC-induced hypersensitive sites observed by DNase I footprinting are starred. B) The binding of purified AsnC protein to a P^32^ end-labelled *cea8* fragment in the presence and absence of L-asparagine (± L-Asn) was investigated using EMSA analysis. The concentration of AsnC in lanes 2–7 and 9–14 was 0.5, 1.05, 2.1, 4.2, 8.4 and 12.6 μM, respectively. The location of free DNA, the position of the wells and the various AsnC/DNA complexes is indicated. C) An *in vitro* DNase I footprint experiment analysing the binding of purified AsnC to the *cea8* promoter. The concentration of AsnC in lanes 2–6 and 8–12 was 0.5, 1.0, 2.1, 4.2, and 6.3 μM, respectively. The AsnC-induced hypersensitive sites are starred (also see panel A) and the location of the different protection patterns observed due to L-asparagine is indicated by red boxes.

The AsnC protein is a member of the Lrp/AsnC family of regulators, which often assemble to form wheel-like octamers and whose DNA binding activity can be modified by small molecules, such as amino acids [13]. AsnC regulates the expression of its own gene, *asnC*, and the *asnA* gene, encoding for a synthetase that catalyses the ammonia-dependent conversion of aspartate to asparagine [14]. To investigate the binding of AsnC to the *cea8* promoter (Fig 3A), we over-expressed and purified the AsnC protein and performed *in vitro* experiments in the presence or absence of the amino acid L-asparagine. EMSA experiments show that several AsnC molecules can interact with *cea8* (Fig 3) and that in the presence of L-asparagine a number of distinct complexes can be observed (Fig 3B). In the absence of L-asparagine, at higher AsnC concentrations, DNA remained in the wells of the gel, indicating that high molecular weight nucleoprotein complexes had formed.

DNase I footprinting was also used to study the location of AsnC binding to the *cea8* promoter sequence, again in the presence or absence of L-asparagine. Results in Fig 3C show that AsnC interacts along the entire length of the 179 bp *cea8* promoter region. Inspection of the *cea8* region, interacting with AsnC, revealed 27 DNase I hypersensitive sites, which is indicative of local bending and distortion of the DNA helix. This results in a widening of the minor groove and makes the DNA more susceptible to DNase I attack, leading to the production of hypersensitive bands [21]. In several locations the presence of L- asparagine altered the binding of AsnC to the *cea8* promoter (see red boxes in Fig 3C). The position of the red boxes in Fig 3C was determined by comparing the AsnC footprint gels in the presence or absence of L-asparagine. Our *in vitro* analysis indicates that AsnC binds to the *cea8* promoter region at multiple sites, likely wrapping the DNA into a complex nucleoprotein assembly, and that the architecture of this complex is altered by the presence of L-asparagine (Fig 3).

### LexA and AsnC can bind to the *cea8* promoter region simultaneously

**Fig 4:**
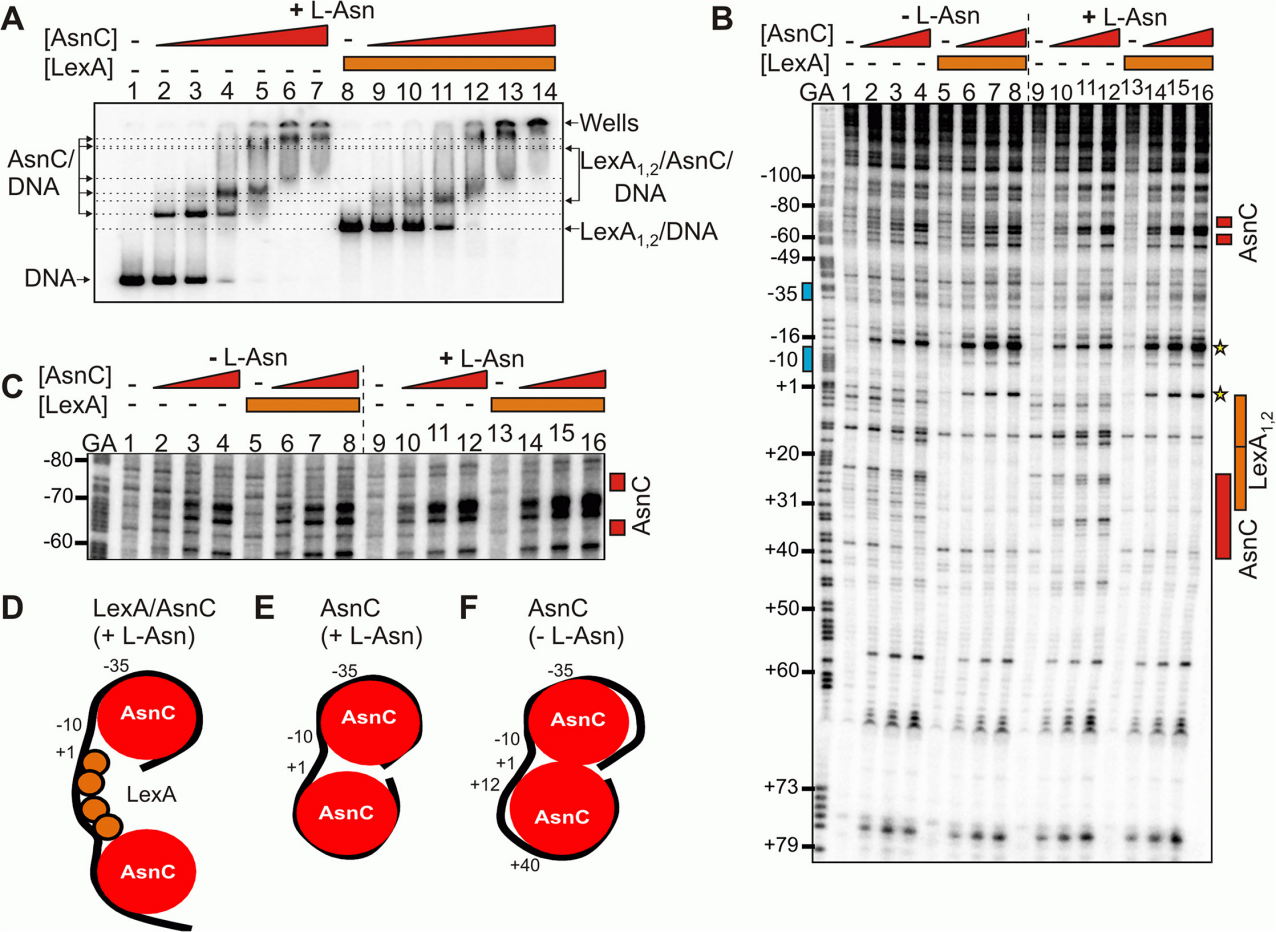
LexA and AsnC can bind to *cea8* promoter region simultaneously. A) The panel shows an EMSA analysing the binding of purified LexA and AsnC protein to a P^32^ end-labelled *cea8* fragment in the presence of L-asparagine (+ L-Asn). The concentration of AsnC in lanes 2–7 and 9–14 was 0.5, 1.05, 2.1, 4.2, 8.4 and 12.6 μM, respectively. LexA protein was present in reactions at concentration of 400 nM. The location of free DNA, the position of the wells and the various protein/DNA complexes is marked. B) The binding of purified LexA and AsnC proteins to the *cea8* promoter fragment was studied by DNase I footprinting in the presence and absence of L-asparagine (± L-Asn). AsnC was included at concentrations of 1.0, 2.1 and 4.2 μM and LexA at a concentration of 400 nM. The prominent hypersensitive bands, corresponding to positions +1 and -16, that are produced by the concurrent binding of AsnC and LexA, are starred. Red boxes indicate the position of AsnC interactions, which are affected by L-Asn. The location of the two LexA binding sites, LexA_1_ and LexA_2_, is indicated by orange boxes and the -10 and -35 promoter elements by blue boxes. C) The panel shows an extended run of the footprint analysis from panel B, focusing on positions -80 to -60 upstream of the transcription start site. Labels are identical to those described above. Panels D-F) show models of the different nucleoprotein complexes formed at the *cea8* promoter by the binding of LexA and AsnC.

Since our data show that AsnC binds at multiple locations and alters the architecture of colicin E8 regulatory region, as well as binding within LexA target sites (Fig 3), we tested if AsnC and LexA can simultaneously bind to the *cea8* promoter region. To investigate this, we performed EMSA analysis on the *cea8* promoter fragment. Using purified LexA and AsnC, in the presence of L-asparagine, we observed a large nucleoprotein complex composed of at least two AsnC functional oligomers, presumably octamers [13], and two LexA dimers interacting at *cea8* (Fig 4A). Note, that LexA was used at a concentration of 400 nM at which LexA repressor occupies both LexA binding sites within *cea8* (Fig 1B). To determine whether occupancy of the DNA by AsnC affects the binding of LexA at the *cea8* promoter region, we performed DNase I footprint analysis and compared signatures of LexA and AsnC in the presence or absence of L-asparagine. In both conditions, LexA repressors bound to tandem targets just downstream of the -10 promoter element (Fig 4B). As observed for AsnC binding in Fig 3C, the addition of L-asparagine also modulated the binding of AsnC in the LexA-AsnC nucleoprotein complex (Fig 4B and 4C). In the AsnC-LexA-*cea8* complex, specific hypersensitive sites were apparent (determined by stars in Fig 4B), suggesting that the binding of both proteins subtly alters the structure or trajectory of the DNA around -10 element (Fig 4D–4F). Thus, we conclude that concurrent binding of LexA and AsnC to the *cea8* regulatory region ensures delayed induction of the DNase E8 synthesis after DNA damage.

### The *cea8* promoter is sensitive to L-asparagine

**Fig 5:**
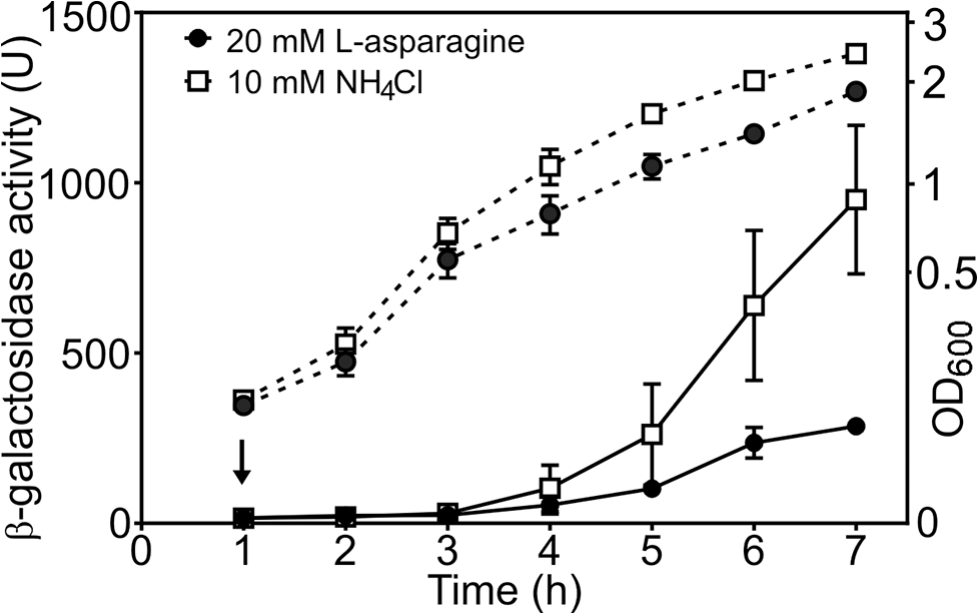
The *cae8* promoter is sensitive to the amino acid L-asparagine. The figure shows measured β-galactosidase activities from wild-type BW25113 cells, carrying the *cea8* promoter region subcloned into pRW50. Cells were grown in M9 minimal medium containing 0.5 mM NH_4_Cl until an OD_600_ of ~0.2, when the culture was split in to two and grown further in the presence of either 10 mM NH_4_Cl or 20 mM L-asparagine. The arrow indicates the time of addition of nalidixic acid and the dashed lines represent OD_600_. Each value is the average of duplicate experiments and the standard deviation is shown.

Our data suggest that tight repression of DNase colicin E8 might be affected by the availability of amino acid L-asparagine and this signal is relayed via AsnC. To test this hypothesis we measured *cea8* promoter activity in SOS-induced wild-type cells grown in the M9 minimal medium containing either 10 mM NH_4_Cl or 20 mM L-asparagine as the sole source of nitrogen. Fig 5 shows that in the L-asparagine containing medium, the expression from *cea8* remains low, whilst it increases in medium containing higher levels of NH_4_Cl. This is in agreement with our *in vitro* data (Figs 3 and 4) and suggests that L-asparagine is needed to stabilize a specific AsnC assembly at the *cea8* promoter region. Hence, we suggest that depletion of L-asparagine is the signal for AsnC de-repression at the *cea8* promoter.

### AsnC regulates the expression of different nuclease colicins

**Fig 6:**
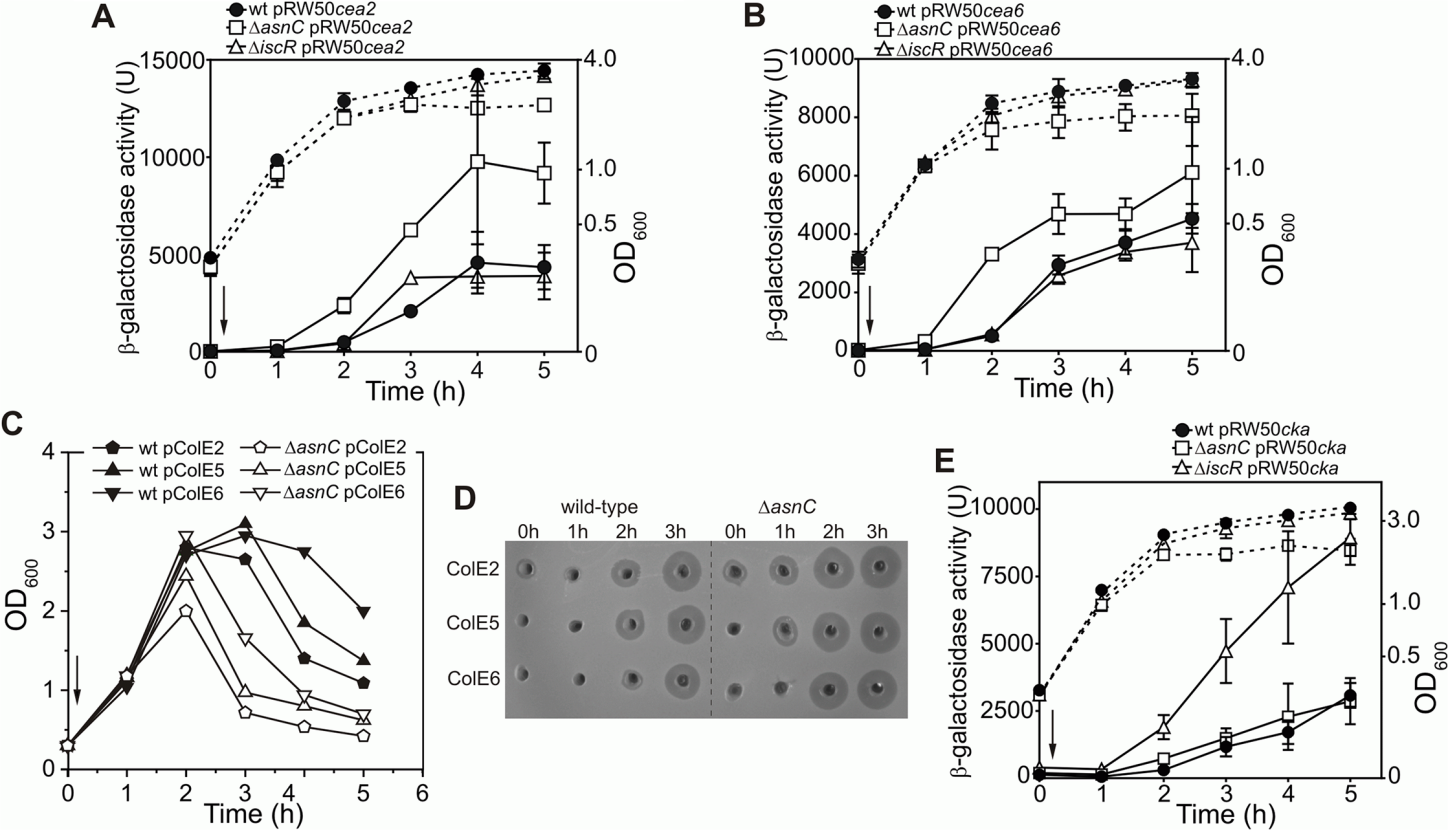
AsnC modulates the temporal induction of different DNA- and RNA-degrading colicins. Expression of the (A) *cea2*::*lacZ* and the (B) *cea6*::*lacZ* fusions in strain BW25113 (wt) and the Δ*iscR* and Δ*asnC* mutants. For panels A and B each value represents the mean ± SD of at least two independent measurements, the arrow indicates the time of the addition of a sub-lethal concentration of nalidixic acid (37 μM) and the dashed lines represent OD_600_. C) Growth curves of BW25113 (wt) and Δ*asnC* cells harbouring naturally occurring plasmids encoding either the DNA degrading colicin E2, the tRNA cleaving colicin E5 or the rRNA cleaving colicin E6. The arrow indicates the time of addition of nalidixic acid. Experiments were performed in duplicate and representative growth curves are shown. D) Assays of colicin production in BW25113 and Δ*asnC* cells, carrying various colicin-encoding plasmids. Equal amounts of cells were collected at hourly time points after the addition of nalidixic acid (0 h) and crude cell extracts were applied into wells in agar plates, overlaid with soft agar harbouring the colicin sensitive strain DH5α pBR322. The experiments were performed in duplicate. E) AsnC protein has little effect on the expression of the pore-forming colicin K. The activity of the *cka* promoter was measured in strain BW25113 (wt) and its Δ*iscR* or Δ*asnC* mutant derivatives. Each value represents the mean ± SD of four independent measurements, the arrow indicates the time of addition of a sub-lethal concentration of nalidixic acid (37 μM) and the dashed lines represent the OD_600_.

To determine whether AsnC modulates the expression of other colicins, we assayed DNase colicin E2 (*cea2*) and rRNase colicin E6 (*cea6*) promoter activities following SOS induction with nalidixic acid using *cea6*::*lacZ* and *cea2*::*lacZ* promoter fusions in the wild-type, Δ*iscR* and Δ*asnC* strains. Results illustrated in Fig 6A and 6B show that the disruption of *asnC* resulted in elevated *cea2* and *cea6* promoter activity immediately after DNA damage induction, when compared to the wild-type and the Δ*iscR* strains. This indicates that AsnC, rather than IscR, is a key transcriptional repressor of the *cea2* and *cea6* promoters. In addition, we transferred the colicinogenic plasmids for these DNase and RNase colicins into the Δ*asnC* and wild-type strains. Following the induction of DNA damage, cell growth (Fig 6C) and colicin production (Fig 6D) was monitored in both strains. In the absence of *asnC*, cells failed to reach as high optical density as in the wild-type strain, suggesting that elevated colicin expression and cell lysis had taken place (Fig 6C). Cell extracts were prepared from cultures before and after DNA damage induction and colicin levels compared by a colicin production bioassay (Fig 6D). After SOS induction, nuclease colicin synthesis was induced earlier for colicins E2, E5 and E6 in the Δ*asnC* strain, in comparison to the wild-type strain, indicating that AsnC directly modulates the expression of a number of other colicin genes. Alignment of the *cea8* promoter region sequence with corresponding sequences from colicin E2, E5 and E6 indicated that these promoters are very similar (S4 Fig) and, thus, similar co-ordinated regulation is perhaps to be expected.

It is clear from S4 Fig that regions of the *cka* promoter are similar to that of *cea8*, particularly around the two LexA SOS boxes. As this region was bound differentially by AsnC at *cea8* in the presence of L-asparagine, we examined whether purified AsnC could bind *in vitro* to a radiolabelled *cka* promoter fragment using EMSA. Results in S5A Fig indicated that AsnC does bind to *cka* and its DNA binding was modulated by L-asparagine. As this raises the possibility that AsnC could regulate colicin K production *in vivo*, we measured *cka* promoter activity after SOS-induction in wild-type, Δ*asnC* and Δ*iscR* cells, carrying a *cka*::*lacZ* fusion cloned into pRW50. Results in Fig 6E indicate that AsnC had little effect on *cka* expression, but confirmed that IscR is a major repressor of the *cka* promoter. In addition, colicin K expression was also examined in our wild-type, Δ*asnC* and Δ*iscR* strains, whilst carrying a colicin K-encoding plasmid (S5B and S5C Fig). These experiments again showed that IscR is the major regulator of colicin K expression and that AsnC has little effect on the expression of this pore-forming colicin.

### Concluding remarks

*E*. *coli* harbours many promoters that are regulated by multiple transcription factors, each of which ensures that different intra- or extracellular signals are integrated into gene expression [22]. At the DNase colicin E8 regulatory region we identified a large nucleoprotein complex composed of two LexA repressors flanked by at least two AsnC octamers, that likely wrap DNA in a nucleosome-like structure to firmly prevent transcription initiation (Fig 4D–4F).

The AsnC protein belongs to the Lrp/AsnC family of transcriptional regulators that are widely distributed among prokaryotes and affect cellular metabolism, often in response to exogenous amino acids [12,13]. In contrast to other members of the family, which are global regulators and affect a variety of bacterial functions [23], the AsnC protein was thought to be a gene specific regulator, controlling only two genes in *E*. *coli* (*asnA* and *asnC*) [14]. Here we report a novel role for AsnC, in which the promoters of the nuclease colicins have “recruited” this protein, enabling regulation in response to L-asparagine levels.

Our data show that an amino acid effector modulates AsnC interaction at the colicin E8 promoter, which influences regulation of *cea8* expression. We predict that, as for the *Neisseria meningitidis* AsnC ortholog, [24], L-asparagine binding modulates the stability of a certain protein oligomeric state and also the mode of binding in the *E*. *coli* AsnC-*cea8-*LexA nucleoprotein complex. Furthermore, as expression of *asnC* is negatively autoregulated and dependent on the Nac protein under nitrogen-limiting conditions [15], nutrient conditions, specifically nitrogen levels, and nitrogen metabolism might coordinate the *cea8* expression through altering the amount of AsnC within the cell. Note that nutrients were recently reported to modulate the release of the DNase colicins by modulating the translation efficiency of the colicin E2 lysis gene transcript [25]. Therefore, AsnC appears to couple metabolic signals to the induction of colicin operon components, in order to synchronise accumulation with the release of the colicin.

In our previous work, we showed that the global transcriptional factor, IscR, in response to the nutritional status of the cell, and, co-dependently with LexA, delays induction of the pore-forming colicin genes after SOS induction [11]. This was a surprising finding as *E*. *coli* IscR had been thought to be primarily involved with controlling housekeeping iron sulphur cluster biogenesis, anaerobic respiration enzymes and biofilm formation [26,27]. Here our data strongly suggests that temporal induction of DNA and RNA targeting colicins is IscR independent, and show that the key regulator is the AsnC repressor. At the *cea8* promoter, AsnC repression seems to reflect L-asparagine levels and presumably serves as an indicator of general amino acid abundance and availability. In contrast, AsnC does not affect the expression of pore-forming colicin K gene expression. Thus, our data imply that the promoters of the nuclease and pore-forming colicins have adopted different transcription regulators to co-ordinately regulate transcription in conjunction with the LexA repressor and distinct metabolic inputs are integrated at these promoters, which both affect the timing and level of colicin induction.

It is clear that colicinogenic plasmids seem to have evolved to exploit transcriptional factors that are of the host origin. We suggest that ubiquitous regulators, present in most *E*. *coli* strains were “picked” in order that the colicinogenic plasmids can be swapped between strains [1], with the colicin promoters being silenced in the same manner. In addition, colicin production and subsequent lysis protein driven colicin release causing death of the producing bacteria, may enable eradication of strains that lack or synthesize a non-functional regulator and cannot efficiently respond or adapt to different environmental signals.

## Material and Methods

### Bacterial strains and plasmids

The bacterial strains, plasmids, promoter fragments and oligodeoxynucleotide primers used in the present study are listed in S3 Table. The *E*. *coli* Keio collection wild-type strain, BW25113, and its derivatives were used throughout the study [16]. To verify the Keio collection deletion strains, a transposon-specific primer Keio1Kn [16] and the gene specific primer (named as gene pre) was used (S3 Table) in 30 cycles of PCR reactions (30 sec 94°C, 30 sec 55°C, 60 sec 72°C). PCR products were analysed on 1.2% agarose gels and stained with ethidium bromide. The colicin D (*cda*), E2 (*cea2*), E5 (*cea5*), E6 (*cea6*), E7 (*cea7*) and E8 (*cea8*) promoter fragments were amplified by PCR from natural colicin encoding plasmids using primers colX_beta_F and colX_beta_R (X denotes the relevant colicin), which introduce flanking EcoRI and HindIII sites (S3 Table). For testing colicin promoter activities, each promoter fragment was cloned into the *lac* expression vector, pRW50. Plasmid constructs were named as pRW50*cxay* (x and y denotes each colicin). As a source of DNA fragments for *in vitro* analysis, EcoRI-HindIII colicin E8 and colicin K promoter fragments were cloned into pSR. To assay colicin synthesis and ensure plasmid selection, the transposon Tn3 (Ap^R^) was inserted into the naturally occurring colicinogenic plasmids of the Pugsley colicin collection [28], harbouring operons for either colicin D, E2, E5, E6, E7 or E8. Strain CL127 carrying Tn3 on the conjugative plasmid pHly152-T8 was used as a donor strain. To generate plasmid pAsnC, for the overexpression of the N-terminal His_6_ AsnC fusion protein, primers asnC_u and asnC_d were used to PCR amplify the *asnC* open reading frame and introduce flanking BamHI and MluI restriction sites. Purified PCR product was subsequently cloned into expression vector pET8c (Novagen) to generate pAsnC.

### Proteins

*E*. *coli* RNA polymerase holoenzyme harbouring σ^70^ (RNAP) was obtained from Epicentre Technologies (Madison). The His_6_-LexA protein was overexpressed and purified as described in [29] and stored in 20 mM Tris (pH 7.3), 200 mM NaCl at -80°C. The His_6_-IscR protein was overexpressed, purified and its concentration determined as described in [11]. To induce the synthesis of AsnC protein, an overnight culture of *E*. *coli* BL21 (DE3)pLysE strain grown on an agar plate, containing ampicilin (100 μg ml^-1^) and chloramphenicol (25 μg ml^-1^), harbouring pAsnC was grown to an optical density at 600 nm (OD_600_) of 0.6 when 0.8 mM isopropyl β-D-1-thiogalactopyranoside (IPTG) was added to the culture. After 4 h of growth the cells were harvested and the N-terminally His_6_-tagged AsnC was affinity purified by Ni-chelate chromatography (Quiagen) and stored at 4°C in 50 mM NaH_2_PO_4_ (pH 8), 300 mM NaCl, 250 mM imidazole. The concentrations of the LexA and AsnC proteins were determined using a NanoDrop 1000 (Thermo Scientific) using the extinction coefficients at 280 nm of 6990 M^-1^ cm^-1^ and of 10555 M^-1^ cm^-1^, respectively.

### β-Galactosidase assays

The low-copy number *lac* expression vector, pRW50 [30], was used to measure the activity of the colicin promoters. Plasmids harbouring colicin promoter fragments (S3 Table) were transformed into the relevant strains. Cells were grown aerobically (180 r.p.m.) at 37°C in Lysogeny Broth (LB) supplemented with tetracycline (12.5 μg ml^-1^). To induce the SOS response, a sub-inhibitory concentration [31], 37 μM, of nalidixic acid (Sigma-Aldrich) was added to the culture when the OD_600_ reached 0.3. Culture samples were assayed for β-galactosidase activity according to the Miller method [32]. The presented values are the averages of at least three independent experiments and are shown with standard deviations. To measure the L-asparagine effect on *cea8* promoter activity, the relevant wild type strain carrying pRW50*cea8* was grown to an OD_600_ ~0.2 in M9 medium [33], containing a low concentration of NH_4_Cl (0.5 mM) to sustain growth. The bacterial culture was then split and supplemented with either 10 mM NH_4_Cl or 20 mM L-asparagine. After the addition of 37 μM nalidixic acid, as indicated, samples were taken and analysed as described above.

### Affinity purification of proteins interacting with the colicin E8 promoter region

A biotinylated 179 bp colicin E8 promoter fragment from position -169 to position +10 from the translation start site (TSS) was generated by PCR using primers Pull_FE8 and Pull_RE8 with pColE8-Tn3 as a template. The DNA fragment was purified by GeneJET PCR purification kit (Thermo Scientific). Immobilisation of the biotinylated DNA (50 μg) to 5 mg of M-280 streptavidin Dynabeads (Invitrogen) was carried out in 15 minutes at room temperature as described [11]. An overnight culture of the *E*. *coli* BW25113, harbouring the pRW50*cea8*, was diluted 1: 200 into 0.5 l LB broth supplemented with tetracycline (12.5 μg ml^-1^) and induced with nalidixic acid (37 μM) once the OD_600_ had reached 0.3. After 45 min, cells were harvested and cell extract prepared as described [31]. Cleared lysates (~20 ml) were mixed with streptavidin beads with or without cross-linked biotinylated *cea8* promoter fragment in 50 ml centrifuge tubes (Costar) and incubated for 10 min with gentle mixing on ice. Dynabeads were collected using a magnet and washed four times in 20 mM Hepes-Na (pH 7.4), 100 mM NaCl, 0.1% (v/v) Tween 20. Proteins were eluted with 500 μl of buffer (20 mM Hepes-Na, 800 mM NaCl, 0.1% (v/v) Tween 20) and concentrated by TCA precipitation. Proteins were resolved on a 12% SDS-PAGE gel (Invitrogen), and visualized by Coomassie blue staining. To identify proteins, nine 1 mm gel slices were excised and analysed by the Functional Genomics, Proteomics and Metabolomics Facility at the University of Birmingham using a Thermo-Finnigan LTW Orbitrap mass spectrometer. Candidate proteins that exhibited DNA binding properties were analysed further.

### Bacteriocin production assays

Colicin synthesis was monitored in the wild-type *E*. *coli* BW25113 strain or its Δ*asnC* derivative JW3721 [16], harbouring one of the colicinogenic plasmids, and was grown aerobically at 37°C in LB broth supplemented with ampicillin. Nalidixic acid (Sigma-Aldrich) was added to the culture at a final concentration of 37 μM, when the OD_600_ reached 0.3. Samples were taken before induction and at 1, 2 and 3 h after. Cells were diluted to obtain 1 ml samples with an OD_600_ of 0.3 and the crude cell extracts were prepared by sonication and the cell debris cleared by centrifugation for 1 min at 17000 x g. 100 μl of each extract was injected into wells in an agar plate containing tetracycline (12.5 μg ml^-1^) overlaid with the lawn of the indicator strain (DH5α harbouring pBR322) as described in [11]. As an alternative approach for colicin determination in the crude cell extracts, 5 μl of a ten-fold or five-fold dilution series of extracts were applied to an agar plate overlaid with the indicator strain as above. Indicator strains were grown at 37°C and the plates photographed using a G:Box (Syngene).

### *In vitro* experiments

EMSA analysis, using purified LexA, IscR, AsnC and RNAP, with the *cea8* and *cka* promoter regions, was performed as described in [34]. DNA fragments were excised from pSR*cea8* or pSR*cka* using EcoRI and HindIII restriction enzymes and purified promotror fragments were labelled at the HindIII end with [γ-32P]-ATP using polynucleotide kinase (NEB). Approximately 0.5 ng of DNA fragment was incubated with varying amounts of purified proteins, as indicated. The reaction buffer contained 20 mM Hepes (pH 8), 5 mM MgCl_2_, 50 mM potassium glutamate, 1 mM DTT, 5% (v/v) glycerol and 0.5 mg/ml BSA and the final reaction volume was 10 μl. Where AsnC was used, the EMSA buffer contained 5 mM L-asparagine (L-asn), where indicated. Samples were incubated at 37°C for 15 min before electrophoresis. For competitive EMSA experiments, DNA fragments were first incubated with various concentrations of LexA (for 15 min at 37°C) followed by the addition of RNAP and incubated for another 15 min at 37°C. Herring sperm DNA was included at a concentration of 6.5 μg ml^-1^ for these experiments. After incubation, all samples were immediately run on a 5% polyacrylamide gel at 12 V cm^-1^ in 0.25 x TBE, running under tension, and were visualised using a Bio-Rad Molecular Imager FX and Quantity One Software (Bio-Rad).

DNase I footprinting of AsnC and LexA at the *cea8* promoter region was performed as described [35], using purified proteins in the presence or absence of 5 mM L-asparagine and a purified EcoRI-HindIII *cea8* fragment that had been ^32^P-end labelled at the HindIII site using polynucleotide kinase and [γ-^32^P]ATP.

## Supporting Information

S1 Fig Expression from colicin promoters is minimal in the absence of nalidixic acid. Measured β-galactosidase activities of strain BW25113, harbouring pRW50, with colicin promoter-*lac* transcriptional fusions. The dashed lines represents OD_600_.
(DOCX)

S2 Fig The effect of colicin expression on bacterial growth. Growth curves of wild-type BW25113 (wt) or Δ*iscR* cells, carrying naturally occurring plasmids, which encode either pore-forming or nuclease colicins. The arrow indicates the time of addition of nalidixic acid.
(DOCX)

S3 Fig Colicin production assay. A ten-fold dilution series of colicin extracts of the wild-type strain BW25113 or the isogenic Δ*iscR* strain, harbouring plasmids encoding pore-forming or nuclease colicins. The indicator strain used was DH5α, harbouring pBR322. Samples were taken before and 1, 2, and 3 h after the induction of the SOS response by nalidixic acid.
(DOCX)

S4 Fig Sequence alignments highlight regulatory elements in the colicin gene promoter regions. The promoter -35 and -10 elements, the SOS box targets, the Shine-Dalgarno sequence and the translation start sites are indicated in red, yellow, blue and orange, respectively. The green box indicates the IscR binding site at the *cka* promoter region. Above the sequences »x« denotes position of the AsnC-induced hypersensitive sites observed by DNAse I footprinting at the colicin E8 promoter region (Fig 3) and the red boxes indicate position of the AsnC interaction affected by L-asn (Fig 4). Nucleotide sequences of the plasmids used in this study were determined by Macrogen (http://dna.macrogen.com/) and were identical to the deposited sequences in GeneBank: ID numbers for colicin K, E2, E5, E6 and E8 are AY929248.1, M29885.1, KF925332.1, M31808.1 and FJ985252.1, respectively.
(DOCX)

S5 Fig IscR and not AsnC is the key regulator of *cka* expression. A) EMSA analysis of the binding of purified AsnC protein to a P^32^ end-labelled colicin K promoter fragment in the presence and absence of L-asparagine (± L-Asn). The concentration of AsnC in lanes 2–7 and 9–14 was 0.5, 1.05, 2.1, 4.2, 8.4 and 12.6 μM, respectively. The location of free DNA, the position of the wells and the various AsnC/DNA complexes is indicated. B) Growth curves of BW25113 (wt) and Δ*asnC* cells harbouring the naturally occurring plasmid, which encodes the pore-forming colicin K (pColK). The arrow indicates the time of addition of nalidixic acid. Experiments were performed in duplicate and representative growth curves are shown. C) Colicin synthesis was measured in BW25113, Δ*asnC* and Δ*iscR* cells carrying a colicin K-encoding plasmid. Cells were collected at hourly time points after the of addition of nalidixic acid (0 h) and a five-fold dilution series of cell extracts were applied on an agar plate supplemented with tetracycline and overlaid with the colicin sensitive strain DH5α pBR322. Results illustrate that in comparison to the colicin K production in the wild-type cells, an hour after SOS induction 5- and 125-times more colicin K is synthesized in the Δ*asnC* and the Δ*iscR* mutant, respectively. The experiments were performed in duplicate and representative results are shown.
(DOCX)

S1 Table Protein candidates identified by mass spectrometry which bound to the *cea8* promoter. The numbering of bands are the same as those indicated in Fig 1; % match – % match to the amino acid sequence of proteins within the *E*. *coli* database using Mascot (Matrix Science) software; MW (kDa) – protein molecular weight.
(DOCX)

S2 Table Screening of potential colicin E8 transcriptional regulators. Overnight cultures of each strain were inoculated 1:100 in 10 ml of LB broth supplemented with tetracyclin (12.5 μg ml^-1^) and 37 μM of nalidixic acid. After 12 h of growth the β-galactosidase activity of the cultures was determined (presented in Miller units (U)). The β-galactosidase ratio is the β-galactosidase value of the *cea8*::*lac* activity observed in each mutant in comparison to the wild-type strain BW25113 (wt). Potential candidates, which were analysed further, are shown in bold.
(DOCX)

S3 Table Bacterial strains, plasmids, promoters and primers used in this study. (DOCX)
