# Supplementary figures and images for: Silencing of DNase Colicin E8 Gene Expression by a Complex Nucleoprotein Assembly Ensures Timely Colicin Induction
Source: PLoS Genet. 2015 Jun 26;11(6):e1005354. doi: 10.1371/journal.pgen.1005354 (PMC4482635; doi:10.1371/journal.pgen.1005354)

**
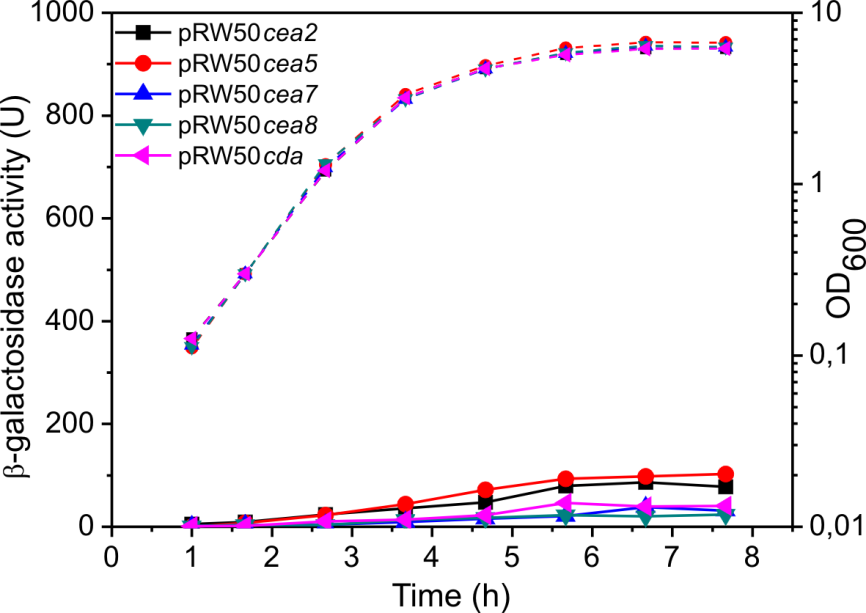
**

**S1 Figure: Expression from colicin promoters is minimal in the absence of nalidixic acid.**

Supplement: S1 Fig — Measured β-galactosidase activities of strain BW25113, harbouring pRW50, with colicin promoter-lac transcriptional fusions. The dashed lines represents OD600. (DOCX) [file pgen.1005354.s001.docx]

**
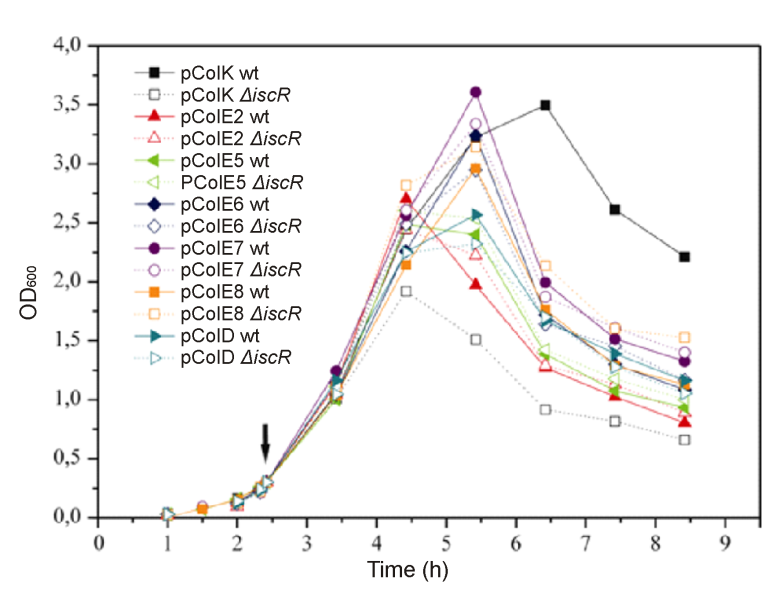
**

**S2 Figure: The effect of colicin expression on bacterial growth.**

Supplement: S2 Fig — Growth curves of wild-type BW25113 (wt) or ΔiscR cells, carrying naturally occurring plasmids, which encode either pore-forming or nuclease colicins. The arrow indicates the time of addition of nalidixic acid. (DOCX) [file pgen.1005354.s002.docx]

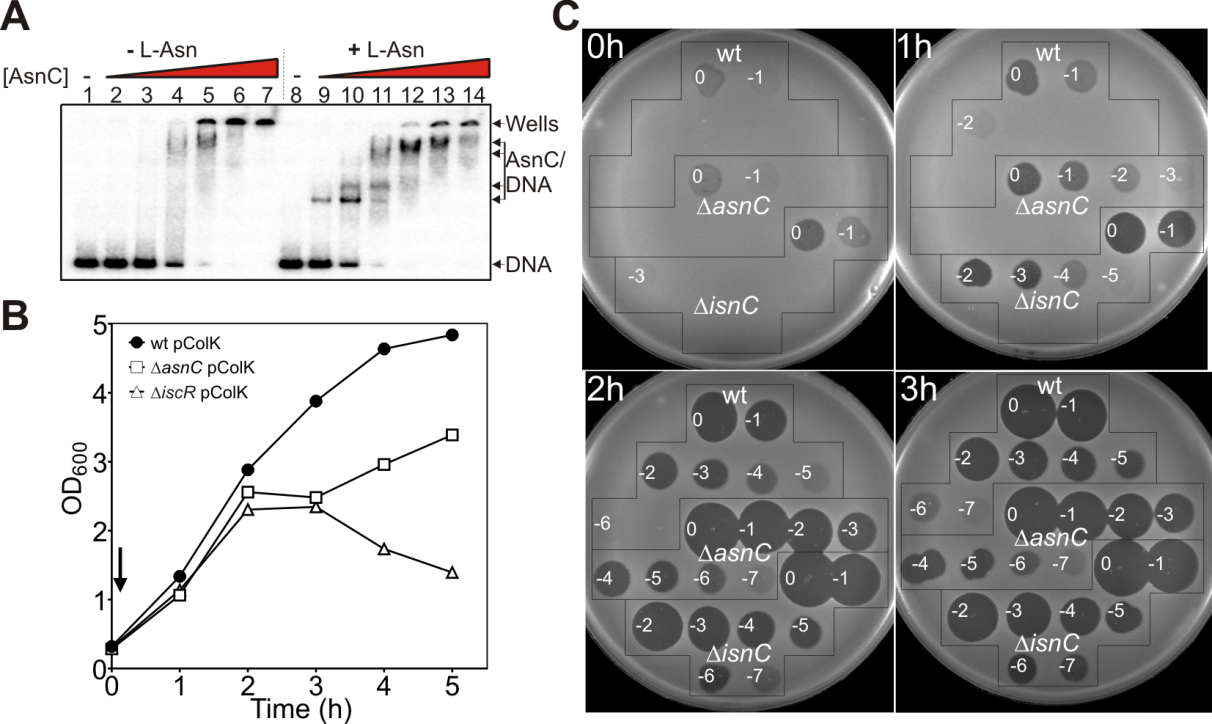


**S5 Figure: IscR and not AsnC is the key regulator of *cka* expression.**

Supplement: S5 Fig — A) EMSA analysis of the binding of purified AsnC protein to a P32 end-labelled colicin K promoter fragment in the presence and absence of L-asparagine (± L-Asn). The concentration of AsnC in lanes 2–7 and 9–14 was 0.5, 1.05, 2.1, 4.2, 8.4 and 12.6 μM, respectively. The location of free DNA, the position of the wells and the various AsnC/DNA complexes is indicated. B) Growth curves of BW25113 (wt) and ΔasnC cells harbouring the naturally occurring plasmid, which encodes the pore-forming colicin K (pColK). The arrow indicates the time of addition of nalidixic acid. Experiments were performed in duplicate and representative growth curves are shown. C) Colicin synthesis was measured in BW25113, ΔasnC and ΔiscR cells carrying a colicin K-encoding plasmid. Cells were collected at hourly time points after the of addition of nalidixic acid (0 h) and a five-fold dilution series of cell extracts were applied on an agar plate supplemented with tetracycline and overlaid with the colicin sensitive strain DH5α pBR322. Results illustrate that in comparison to the colicin K production in the wild-type cells, an hour after SOS induction 5- and 125-times more colicin K is synthesized in the ΔasnC and the ΔiscR mutant, respectively. The experiments were performed in duplicate and representative results are shown. (DOCX) [file pgen.1005354.s005.docx]
