# Supplementary material for: Silencing of DNase Colicin E8 Gene Expression by a Complex Nucleoprotein Assembly Ensures Timely Colicin Induction
Source: PLoS Genet. 2015 Jun 26;11(6):e1005354. doi: 10.1371/journal.pgen.1005354 (PMC4482635; doi:10.1371/journal.pgen.1005354)
Supplement: S3 Fig — A ten-fold dilution series of colicin extracts of the wild-type strain BW25113 or the isogenic ΔiscR strain, harbouring plasmids encoding pore-forming or nuclease colicins. The indicator strain used was DH5α, harbouring pBR322. Samples were taken before and 1, 2, and 3 h after the induction of the SOS response by nalidixic acid. (DOCX) [file pgen.1005354.s003.docx]

**S3 Figure: Colicin production assay.**
