# Supplementary material for: Silencing of DNase Colicin E8 Gene Expression by a Complex Nucleoprotein Assembly Ensures Timely Colicin Induction
Source: PLoS Genet. 2015 Jun 26;11(6):e1005354. doi: 10.1371/journal.pgen.1005354 (PMC4482635; doi:10.1371/journal.pgen.1005354)
Supplement: S4 Fig — The promoter -35 and -10 elements, the SOS box targets, the Shine-Dalgarno sequence and the translation start sites are indicated in red, yellow, blue and orange, respectively. The green box indicates the IscR binding site at the cka promoter region. Above the sequences »x« denotes position of the AsnC-induced hypersensitive sites observed by DNAse I footprinting at the colicin E8 promoter region (Fig 3) and the red boxes indicate position of the AsnC interaction affected by L-asn (Fig 4). Nucleotide sequences of the plasmids used in this study were determined by Macrogen (http://dna.macrogen.com/) and were identical to the deposited sequences in GeneBank: ID numbers for colicin K, E2, E5, E6 and E8 are AY929248.1, M29885.1, KF925332.1, M31808.1 and FJ985252.1, respectively. (DOCX) [file pgen.1005354.s004.docx]

**S4 Figure: Sequence aligments highlight regulatory elements in colicin gene promoter regions.**
