# Supplementary material for: Silencing of DNase Colicin E8 Gene Expression by a Complex Nucleoprotein Assembly Ensures Timely Colicin Induction
Source: PLoS Genet. 2015 Jun 26;11(6):e1005354. doi: 10.1371/journal.pgen.1005354 (PMC4482635; doi:10.1371/journal.pgen.1005354)
Supplement: S1 Table — The numbering of bands are the same as those indicated in Fig 1; % match – % match to the amino acid sequence of proteins within the E. coli database using Mascot (Matrix Science) software; MW (kDa) – protein molecular weight. (DOCX) [file pgen.1005354.s006.docx]

**S1 Table:** **Protein candidates identified by mass spectrometry which bound to the *cea8* promoter.**

| **Band #** | **Protein dscription** | **% match** | **MW (kDa)** |
| --- | --- | --- | --- |
| 1 | **yohL** gene product [*Escherichia coli* O157:H7 str. EDL933] (synonim **rcnR**) | 76,67 | 10,1 |
|  | transcriptional activator **hlyU** [*Escherichia coli* 3431] | 39,71 | 7,3 |
| 2 | DNA-binding transcriptional repressor **ArsR** [*Shigella flexneri* 5 str. 8401] | 15,38 | 13,3 |
| 3 | DNA-binding protein **stpA** [*Escherichia coli* 1827-70] | 75,57 | 15,0 |
|  | DNA-binding transcriptional regulator **AsnC** [*Escherichia coli* OP50] | 36,91 | 16,5 |
| 4 | DNA starvation/stationary phase protection protein **Dps** [*Escherichia coli* CFT073] | 97,01 | 18,7 |
|  | iscR transcriptional regulator **IscR** transcriptional dual regulator [*Shigella flexneri* 2930-71] | 74,50 | 15,9 |
| 5 | transcriptional regulator **YbjK** [*Escherichia coli* 536] | 10,67 | 20,4 |
| 6 | **LexA** repressor [*Escherichia coli* O157:H7 EDL933] | 88,12 | 22,3 |
|  | putative GTP-binding protein **EngB** [*Escherichia coli* EPECa12] | 48,99 | 22,2 |
| 7 | **dpiA**-phosphorylated DNA binding transcriptional dual regulator [*Escherichia coli* DEC5A] (synonim **citB**) | 50,23 | 24,7 |
|  | DNA-binding transcriptional repressor **LldR** [*Escherichia coli* O104:H4 str. 01-09591] | 32,47 | 21,9 |
|  | galactonate operon transcriptional repressor [*Escherichia coli* MS 79-10] **dgoR** | 27,31 | 25,8 |
| 8 | repressor of blue light-responsive genes [*Escherichia coli* str. K-12 substr. MG1655] **ycgE** | 67,08 | 28,2 |
|  | **ompR** gene product [*Escherichia coli* O157:H7 str. EDL933] | 71,13 | 27,3 |
|  | **GntR** family transcriptional regulator [*Escherichia coli* E24377A] | 52,42 | 28,2 |
|  | **sfsA** gene product [*Escherichia coli* O157:H7 str. EDL933] | 37,18 | 26,2 |
|  | DNA-binding transcriptional regulator **FrlR** [*Escherichia coli* E24377A] | 28,40 | 27,8 |
|  | DNA-binding transcriptional repressor **MngR** [*Escherichia coli* HS] | 40,42 | 28,2 |
|  | **cpxR** gene product [*Escherichia coli* O157:H7 str. EDL933] | 27,59 | 26,3 |
|  | **TorR** [*Escherichia coli* K-12] | 23,04 | 26,2 |
|  | **glpR** transcriptional repressor [*Escherichia coli* DEC10E] | 12,86 | 26,2 |
| 9 | DNA-binding transcriptional repressor **DeoR** [*Escherichia coli* 536] | 36,51 | 28,5 |
|  | **yihW** gene product [*Escherichia coli* O157:H7 str. EDL933] | 55,39 | 29,5 |
|  | transcriptional regulator **AllR** [*Escherichia coli* SMS-3-5] | 29,69 | 27,6 |
|  | DNA-bindng transcriptional repressor [*Escherichia coli* str. K-12 substr. MG1655] **srlR** | 35,41 | 28,2 |
|  | putative DNA-binding transcriptional regulator [*Escherichia coli* ED1a] **yegW** | 46,77 | 28,3 |
|  | DNA-binding transcriptional regulator **GlcC** [*Escherichia coli* 536] | 28,74 | 28,8 |
|  | **ydjF** gene product [*Escherichia coli* P12b] | 35,83 | 26,9 |
|  | DNA-binding transcriptional activator **FucR** [*Escherichia coli* O157:H7 EDL933] | 20,58 | 27,3 |
