# Supplementary material for: Silencing of DNase Colicin E8 Gene Expression by a Complex Nucleoprotein Assembly Ensures Timely Colicin Induction
Source: PLoS Genet. 2015 Jun 26;11(6):e1005354. doi: 10.1371/journal.pgen.1005354 (PMC4482635; doi:10.1371/journal.pgen.1005354)
Supplement: S2 Table — Overnight cultures of each strain were inoculated 1:100 in 10 ml of LB broth supplemented with tetracyclin (12.5 μg ml-1) and 37 μM of nalidixic acid. After 12 h of growth the β-galactosidase activity of the cultures was determined (presented in Miller units (U)). The β-galactosidase ratio is the β-galactosidase value of the cea8::lac activity observed in each mutant in comparison to the wild-type strain BW25113 (wt). Potential candidates, which were analysed further, are shown in bold. (DOCX) [file pgen.1005354.s007.docx]

**S2 Table: Screening of potential colicin E8 transcriptional regulators.**

| **Strain characteristics** | **β-galactosidase activity (U)** | **β-galactosidase ratio** |
| --- | --- | --- |
| *ΔallrR* pRW50*cea8* | 74 | 1,0 |
| ***ΔasnC*** pRW50*cea8* | **225** | **3,2** |
| *ΔglpR* pRW50*cea8* | 117 | 1,6 |
| ***ΔstpA*** pRW50*cea8* | **234** | **3,3** |
| *ΔglcC* pRW50*cea8* | 105 | 1,5 |
| *ΔarsR* pRW50*cea8* | 107 | 1,5 |
| *ΔlldR* pRW50*cea8* | 135 | 1,9 |
| *ΔdgoR* pRW50*cea8* | 99 | 1,4 |
| ***ΔompR*** pRW50*cea8* | **213** | **3,0** |
| *ΔydjF* pRW50*cea8* | 121 | 1,7 |
| *ΔtorR* pRW50*cea8* | 148 | 2,1 |
| *ΔfrlR* pRW50*cea8* | 185 | 2,6 |
| *ΔgntR* pRW50*cea8* | 86 | 1,2 |
| ***ΔygbI*** pRW50*cea8* | **219** | **3,1** |
| *ΔdgsA* pRW50*cea8* | 110 | 1,5 |
| ***ΔybjK*** pRW50*cea8* | **344** | **4,8** |
| *ΔdpiA* pRW50*cea8* | 49 | 0,7 |
| *ΔycgE* pRW50*cea8* | 134 | 1,9 |
| *ΔyohL* pRW50*cea8* | 176 | 2,5 |
| *ΔhlyU* pRW50*cea8* | 82 | 1,2 |
| *ΔfucR* pRW50*cea8* | 85 | 1,2 |
| *ΔcpxR* pRW50*cea8* | 62 | 0,9 |
| *ΔdeoR* pRW50*cea8* | 91 | 1,3 |
| *ΔsfsA* pRW50*cea8* | 73 | 1,0 |
| ***ΔyihW*** pRW50*cea8* | **222** | **3,1** |
| ***ΔyegW*** pRW50*cea8* | **248** | **3,5** |
| *ΔsrlR* pRW50*cea8* | 95 | 1,3 |
| ***ΔmngR*** pRW50*cea8* | **542** | **7,6** |
| *Δdps* pRW50*cea8* | 117 | 1,6 |
| wt pRW50*cea8* | 71 | 1,0 |
| *ΔiscR* pRW50*cea8* | 115 | 1,6 |
