# Supplementary material for: Silencing of DNase Colicin E8 Gene Expression by a Complex Nucleoprotein Assembly Ensures Timely Colicin Induction
Source: PLoS Genet. 2015 Jun 26;11(6):e1005354. doi: 10.1371/journal.pgen.1005354 (PMC4482635; doi:10.1371/journal.pgen.1005354)
Supplement: S3 Table — (DOCX) [file pgen.1005354.s008.docx]

S3 Table: Bacterial strains, plasmids, promoters and primers used in this study.

|  | **Relevant characteristics** | | | **Reference or source** | |
| --- | --- | --- | --- | --- | --- |
| ***E. coli* K12 strains** | | | | | |
| DH5α | | *thi-1 hsdR17 gyrA96 recA1 endA1 glnV44 relA1* Φ80d*lacZ*ΔM15 *phoA8* | Life Technologies | | |
| BW25113 | | *rrnB3* Δ*lacZ4787 hsdR514* Δ(*araBAD*)*567*  Δ(*rhaBAD)568 rph-1* | [1] | | |
| BL21(DE3)pLysE | | F^–^ *dcm lon ompT hsdS*(r_B_^-^ m_B_^-^) *gal* λ(DE3) pLysE (Cm^R^) | Invitrogen | | |
| JW3721 | | BW25113 Δ*asnC* | [1] | | |
| JW2515 | | BW25113 Δ***iscR*** | [1] | | |
| JW5567 | | BW25113 Δ***yihW*** | [1] | | |
| JW3386 | | BW25113 Δ***glpR*** | [1] | | |
| JW0824 | | BW25113 Δ***deoR*** | [1] | | |
| JW0494 | | BW25113 Δ***allR*** | [1] | | |
| JW2676 | | BW25113 Δ***srlR*** | [1] | | |
| JW1149 | | BW25113 Δ***ycgE*** | [1] | | |
| JW1586 | | BW25113 Δ***dgsA*** | [1] | | |
| JW2947 | | BW25113 Δ***glcC*** | [1] | | |
| JW1759 | | BW25113 Δ***ydjF*** | [1] | | |
| JW5698 | | BW25113 Δ***frlR*** | [1] | | |
| JW2776 | | BW25113 Δ***fucR*** | [1] | | |
| JW2088 | | BW25113 Δ***yegW*** | [1] | | |
| JW0142 | | BW25113 Δ***sfsA*** | [1] | | |
| JW5946 | | BW25113 Δ***gntR*** | [1] | | |
| JW3579 | | BW25113 Δ***lldR*** | [1] | | |
| JW0719 | | BW25113 Δ***mngR*** | [1] | | |
| JW5114 | | BW25113 Δ***ybjK*** | [1] | | |
| JW3368 | | BW25113 Δ***ompR*** | [1] | | |
| JW2642 | | BW25113 Δ*hlyU* | [1] | | |
| JW2092 | | BW25113 Δ*yohL* | [1] | | |
| JW3468 | | BW25113 Δ***arsR*** | [1] | | |
| JW2705 | | BW25113 Δ***ygbI*** | [1] | | |
| JW0612 | | BW25113 Δ*dpiA* | [1] | | |
| JW3883 | | BW25113 Δ***cpxR*** | [1] | | |
| JW5627 | | BW25113 Δ***dgoR*** | [1] | | |
| JW0797 | | BW25113 Δ***dps*** | [1] | | |
| JW0980 | | BW25113 Δ***torR*** | [1] | | |
| JW2644 | | BW25113 Δ***stpA*** | [1] | | |
| **Plasmids** | |  |  | | |
| pKCT1 | | *TaqI* fragments with pUC19 Ap^R^ gene cloned into pColK‑K235, encoding colicin K | [2] | | |
| pSR | | pBR322 derivative containing a λ *oop* transcription terminator | [3] | | |
| pRW50 | | Broad-host-range *lacZ* fusion vector for cloning promoters on EcoRI HindIII fragments; contains the RK2 origin of replication, Tc^R^ | [4] | | |
| pRW50*cea2* | | pRW50 with *ce2a* promoter fragment | This study | | |
| pRW50*cea5* | | pRW50 with *ce5a* promoter fragment | This study | | |
| pRW50*cea6* | | pRW50 with *ce6a* promoter fragment | This study | | |
| pRW50*cea7* | | pRW50 with *ce7a* promoter fragment | This study | | |
| pRW50*cea8* | | pRW50 with *cea8* promoter fragment | This study | | |
| pRW50*cda* | | pRW50 with *cda* promoter fragment | This study | | |
| pSR*cea8* | | pSR with *cea8* promoter fragment | This study | | |
| pSR*cka* | | pSR with *cka* promoter fragment | [5] | | |
| pET8c | | Expression plasmid, N-terminal His tag, Ap^R^ | Novagen | | |
| pAsnC | | IPTG inducible derivative of the pET8c for expression of N‑terminaly His‑tagged AsnC | This study | | |
| pColD-CA23 | | Colicin D operon (*cda cdi cdl*) | [6] | | |
| pColE2-P9 | | Colicin E2 operon (*ce2a ce2i ce2l*) | [6] | | |
| pColE5-099 | | Colicin E5 operon (*ce5a ce5i ce5l*) | [6] | | |
| pColE6-CT14 | | Colicin E6 operon (*ce6a ce6i ce6l*) | [6] | | |
| pColE7-K317 | | Colicin E7 operon (*ce7a ce7i ce7l*) | [6] | | |
| pPC101 | | Colicin E8 operon (*cea8 ce8i ce8l*) | [6] | | |
| pColE2 | | pColE2‑P9 with Tn3, Ap^R^ | This study | | |
| pColE5-Tn3 | | pColE5-099 with Tn3, Ap^R^ | This study | | |
| pColE6-Tn3 | | pColE6-CT14 with Tn3, Ap^R^ | This study | | |
| pColE7-Tn3 | | pColE7-K317 with Tn3, Ap^R^ | This study | | |
| pColE8-Tn3 | | pPC101 with Tn3, Ap^R^ | This study | | |
| pColA | | Encoding colicin A, Ap^R^ | [7] | | |
| pColE1 | | Encoding colicin E1, Ap^R^ | [7] | | |
| pColN | | Encoding colicin N, Ap^R^ | [7] | | |
| pBR322 | | Tc^R^, Ap^R^ | [8] | | |
| **Promoters** | | (all on EcoRI HindIII fragments) |  | | |
| *cea8* | | pPC101 *cea8* promoter fragment carrying nucleotide sequences from ‑381 to +14 from the translation start site (TSS) |  | | |
| *cea7* | | pColE7-K317 *cea7* promoter fragment carrying nucleotide sequences from ‑266 to +17 from the TSS |  | | |
| *cea6* | | pColE6-CT14 *cea6* promoter fragment carrying nucleotide sequences from ‑332 to +16 from the TSS |  | | |
| *cea5* | | pColE5-099 *cea5* promoter fragment carrying nucleotide sequences from ‑332 to +16 from the TSS |  | | |
| *Ccea2* | | pColE2‑P9 *cea2* promoter fragment carrying nucleotide sequences from ‑332 to +16 from the TSS |  | | |
| *cda* | | pColD-CA23 *cda* promoter fragment carrying nucleotide sequences from ‑383 to +17 from the TSS |  | | |
| **Primers** | | Restriction sites are underlined, Btn denotes the biotin label. Sequences are all shown 5`‑3`. | | |  |
| Pull_FE8 | | [Btn]GCTCTGCGTTTTCTAAGTGTTATC | | |  |
| Pull_RE8 | | caccgctcataaaagtccctcttt | | |  |
| colD_beta_F | | CTCGAATTCGCGTCGCTTTGTTTCCT | | |  |
| colD_beta_R | | CCCAAGCTTCCTTCGTAATCACTCATA | | |  |
| colE2/E5/E6_beta_F | | CTCGAATTCTCAACTCGGTTTTAATCAGA | | |  |
| colE2/E5/E6_beta_R | | CCTAAGCTTCATCGCCACCGCTCATAA | | |  |
| colE7_beta_F | | CTCGAATTCTAAACTGGCGGAGAAACCTGT | | |  |
| colE7_beta_R | | CTCAAGCTTCCATCTCCACCGCTCATAAA | | |  |
| colE8_beta_F | | CTCGAATTCCTTTTTGCTGCCATCAACA | | |  |
| colE8_beta_R  asnC_u  asnC_d  Keio1Kn  iscR P1  asnC pre  yihW pre  glpR pre  deoR pre  allR pre  srlR pre  ycgE pre  dgsA pre  glcC pre  ydjF pre  frlR pre  fucR pre  gntR pre  lldR pre  mngR pre  ybjK pre  ompR pre  hlyU pre  yohL pre  arsR pre  dpiA pre  cpxR pre  dps pre  torR pre  stpA pre | | CTCAAGCTTTCTCCACCGCTCATAAAAG  CGCGGATCCGAAAATTATCTGATCGACAATCTG  CGCACGCGTtcaGGGCTTGATGGTACGCATG  CAGTCATAGCCGAATAGCCT  CGCATCCGACAACAGGTAC  ctgcgtcctgttgcttatga  gcaacaagtggggatttagc  TCCGCAAAGTGGCATTTAC  ggattgctggcaggtatgat  gtctggcatcacccactttt  agcccgatgtgatctttcc  tcagcacattctttcacatgatt  tgctctcaggtgagggaa  gggcttttcgttttcgtcta  cagacgcaacattgatgagg  ggcgaaaaccattcagtacc  ctgacatcatccgcatcaat  cgtgctgatgtacgcttcc  gatgatctccccgcaatcta  tgggtcagagtcgtcagattc  ccaggacgccattaacag  cgcacattgggtataacgtg  ctccagaaacccatatgtactcc  gatgaagaaccaggcgtttc  cgtcctgattcagacctcctt  gagcagggggtcagtacg  ctgcaaacatgcgtcagg  tggcgagcagataaataagaa  cgtagcgcgttccagagt  gccgctctgttcaccaat | | |  |

1. Baba T, Ara T, Hasegawa M, Takai Y, Okumura Y, Baba M, et al. Construction of *Escherichia coli* K-12 in-frame, single-gene knockout mutants: the Keio collection. Mol Syst Biol. 2006;2: 2006 0008.

2. Mulec J, Podlesek Z, Mrak P, Kopitar A, Ihan A, Žgur-Bertok D. A *cka*-*gfp* transcriptional fusion reveals that the colicin K activity gene is induced in only 3 percent of the population. J Bacteriol. 2003;185: 654-659.

3. Kolb A, Kotlarz D, Kusano S, Ishihama A. Selectivity of the *Escherichia coli* RNA polymerase E sigma 38 for overlapping promoters and ability to support CRP activation. Nucleic Acids Res. 1995;23: 819-826.

4. Lodge J, Fear J, Busby S, Gunasekaran P, Kamini NR. Broad host range plasmids carrying the *Escherichia coli* lactose and galactose operons. FEMS Microbiol Lett. 1992;74: 271-276.

5. Butala M, Sonjak S, Kamenšek S, Hodošček M, Browning DF, Žgur-Bertok D, et al. Double locking of an *Escherichia coli* promoter by two repressors prevents premature colicin expression and cell lysis. Mol Microbiol. 2012;86: 129-139.

6. Pugsley AP. The ins and outs of colicins. Part I: Production, and translocation across membranes. Microbiol Sci. 1984;1: 168-175.

7. Jerman B, Butala M, Žgur-Bertok D. Sublethal concentrations of ciprofloxacin induce bacteriocin synthesis in *Escherichia coli*. Antimicrob Agents Chemother. 2005;49: 3087-3090.

8. Bolivar F, Rodriguez RL, Greene PJ, Betlach MC, Heyneker HL, Boyer HW, et al. Construction and characterization of new cloning vehicles. II. A multipurpose cloning system. Gene. 1977;2: 95-113.
